# Exo-circRNAs: a new paradigm for anticancer therapy

**DOI:** 10.1186/s12943-019-0986-2

**Published:** 2019-03-30

**Authors:** Hetian Bai, Kexin Lei, Fei Huang, Zhou Jiang, Xikun Zhou

**Affiliations:** 1 National Clinical Research Center for Geriatrics and State Key Laboratory of Biotherapy, Cancer Center, West China Hospital, West China Medical School, Sichuan University, and Collaborative Innovation Center for Biotherapy, Chengdu, China; 2 State Key Laboratory of Oral Diseases, National Clinical Research Center for Oral Diseases, Chinese Academy of Medical Sciences Research Unit of Oral Carcinogenesis and Management, West China Hospital of Stomatology, Sichuan University, Chengdu, China

**Keywords:** CircRNAs, Exosomes, Exo-circRNAs, Cancer therapy

## Abstract

CircRNAs, as new members of long noncoding RNAs, have been the focus of recent investigation. CircRNAs feature a closed continuous loop structure without 5′-3′ polarity or a poly A tail. Many studies have reported the potential application of circRNAs in the clinic as new biomarkers and therapeutic targets in different diseases, especially for cancer. Additionally, the exosomes are important vehicles in cell-to-cell communication. And exo-circRNAs are circRNAs in exosomes which can be detected to provide additional evidence for conventional diagnostic methods and can be applied to suppress the malignant progress in cancer. In this review, we describe the biogenesis, characteristics, and functions of circRNAs and exosomes. Specifically, we present a comprehensive update of the promising role of exo-circRNAs in anticancer therapy.

## Introduction

CircRNAs (circular RNAs) are a kind of abundant and widespread noncoding RNA that universally exist in eukaryotic cells and regulate gene expression [1, 2]. The diverse biological functions of circRNAs are being studied widely. Among them, the most striking function is acting as a miRNA sponge—circRNAs can bind to single or multiple miRNAs and regulate the expression of their downstream genes [3–5]. Moreover, circRNAs feature tissue- and developmental stage-specific expression. Consequently, these molecules are expected to be extracted from clinical samples and analyzed, and studies on the potential of circRNAs to become biomarkers have recently been undertaken widely.

Closely linked to circRNAs, exosomes are another hotspot in recent years. Exosomes are nanoscale membrane vesicles that can be generated from most cell types. As we know, intercellular information transmission is crucial for tumor progression in the tumor microenvironment, and this is the main function of exosomes [6]. These molecules can be secreted into body fluid, such as blood, urine and saliva, with various components like RNAs, proteins, and even DNAs, which is followed by delivering their cargoes to adjacent cells and influence cell biological behaviors. A recent study indicated that circRNAs are abundant and stable in exosomes and can continually play their roles after the exosomes are taken up by neighboring cells [7]. In this review, we begin with the characteristics, origination and function of circRNAs and exosomes. Particularly, we illustrate the research progress of exosomal circRNAs (exo-circRNAs) in cancer and highlight the application of them in anticancer treatment.

## CircRNAs: features, biogenesis and functions

Early in 1976, circRNAs were first identified in RNA viruses via electron microscopy [8, 9]. However, little attention has been paid to the exploitation of their value because these molecules were deemed to be the product of error splicing for over two decades [10, 11]. Until recent years, the potential significance of circRNAs has gradually been discovered, and researchers have begun to determine the properties, biogenesis and functions of circRNAs.

CircRNAs feature stability, abundance, prevalence and conservation [12]. Intriguingly, unlike other RNAs, the absence of 5′ caps and 3′ tails enables circRNAs to form resistance to RNases, which results in their higher stability compared with linear RNAs [13]. Consequently, circRNAs may accumulate in cells to influence pathological processes, such as neurological diseases, and the clearance mechanism of circRNAs is still being explored [14, 15]*.* The richness of circRNAs has also been confirmed—to date, over 100,000 types of circRNAs have been derived, revealing their abundance [16]. Moreover, with the broad application of RNA sequencing (RNA-Seq), the expression of circRNAs is widely detected in various species, including humans, mice, plants, fruit flies, fungi and many other organisms [17–20]. The conservation of circRNAs is mainly presented as the shared expression of circRNAs between mammals. For example, approximately 5–10% of circRNAs in the human brain can also be expressed in the porcine brain [21]. Taken together, these characteristics make circRNAs valuable as biomarkers or therapeutic targets in the clinic.

Depending on the source of the generation, there are mainly four kinds of circRNAs—exonic circRNAs (ecircRNAs) [22], intronic circRNAs (ciRNAs) [23], exonic-intronic circRNAs (EIciRNAs) [24] and circRNAs generated from tRNAs (tricRNAs) [25]. Studies have shown that the dominance of circRNAs is generated from exons, and the formation of circRNAs usually involves the following two steps. First, the upstream intron of one or more exon pairs and the downstream intron fit together. Then, the 2′ hydroxyl of the upstream intron reacts with the 5′ phosphate of the downstream intron. Afterwards, the 3′ hydroxyl of the 3′ exon reacts with the 5′ phosphate of the 5′ exon, and a circRNA is finally formed [12].

Growing evidences have confirmed that circRNAs are involved in physiological and pathological processes that are closely related to their biological functions. In summary, we can assign the functions of circRNAs into the following categories: regulate linear RNA transcription, sponge miRNAs, sponge proteins, interact with proteins and translate to proteins. Moreover, the most extensively studied function of circRNAs is as miRNA sponges [3–5]. MiRNAs are well-known competitive endogenous RNAs (ceRNAs) with miRNA response elements (MREs) and can be combined with downstream mRNAs to reduce their expression [26, 27]. CircRNAs containing complementary sequences bind to corresponding miRNAs, suggesting a potential role in mediating mRNAs expression. For instance, the murine sex-determining region Y (SRY) harboring 16 binding sites can sponge miR-138 and regulate the downstream mRNA [3]. Consequently the sponge effect of circRNAs takes part in many disease-related pathways and is worthy of further study.

## Exosomes: origination, biogenesis and functions

Exosomes are a class of 40–150 nm extracellular vehicles (EVs) generated and released by most cells [28], such as T cells, B cells, dendritic cells, and mast cells. Exosomes bud directly from the plasma membrane, and on their surface, there are various biomolecules, including RNA, lipids, proteins, and possibly DNA [29]. Inside exosomes, DNA, mRNA, miRNA, and different proteins exist.

Exosomes derive from the endosome pathway [30]. Upon early to late endosome maturation, multivesicular bodies (MVBs) are formed by the special inward budding of the endosome. MVBs can fuse with lysosomes, and the intraluminal vesicles (ILVs) inside undergo degradation. When MVBs fuse with the cell membrane, another inward budding takes place in ILVs, generating nanosized vesicles and secreting these molecules to the extracellular space, which are called exosomes. The Endosomal Sorting Complex Required for Transport (ESCRT) machinery plays an essential role in promoting the formation of endosomes [31, 32]. ESCRT0 recognizes and obtains ubiquitinated proteins in the late endosome membrane. ESCRT1 and ESCRT2 both trigger the budding of MVBs and the sorting of proteins into exosomes. After which ESCRT3 forms a spiral-shaped structure that contributes to the stegnosis of the budding neck of MVBs, and then the ATPase Vps 4 drives membrane scission. At the end of the process, vacuolar protein sorting 4 (Vps 4) mediates the recycling of all ESCRT molecules. The ubiquitinated protein functions in modifying or regulating the localization and function of ESCRT [33, 34]. The secretion of exosomes is regulated by different molecules, for example, Rab27 [35], Rab35 [36], and Ral proteins.

**Fig. 1:**
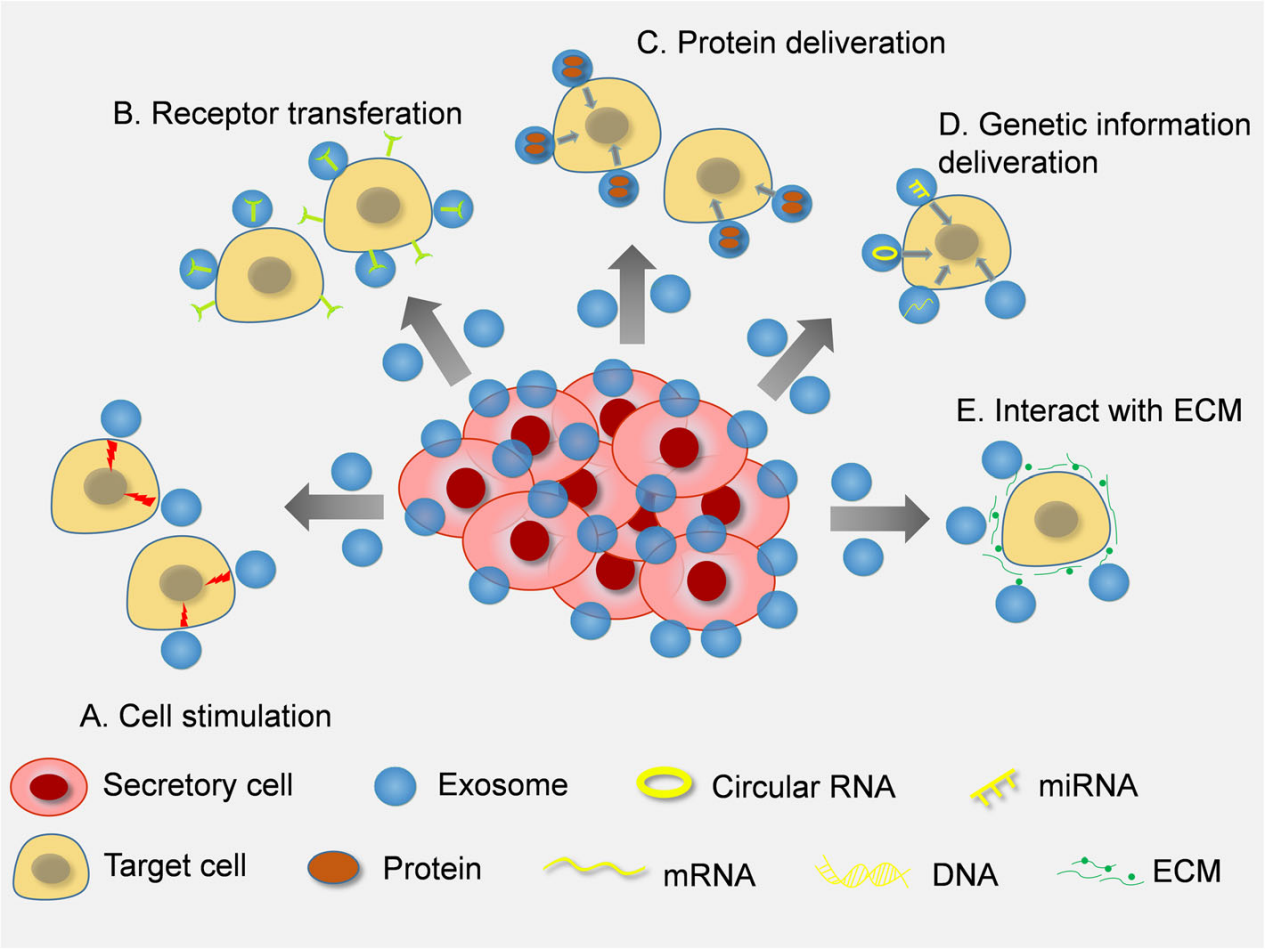
Functions of exosomes in the microenvironment. **a** Exosomes can promote cell activities through message transfer. **b** The receptors could be important cargoes from cell to cell like platelets, endothelial cells, and tumor cells. **c** Proteins in exosomes would be released in target cells and alter cell activities. **d** Cell-to-cell genetic information transfer can be led by exosomes containing genetic materials, such as mRNAs, circRNAs, and miRNAs. **e** Exosomes can bind to ECM and trigger some cell activities

In recent years, exosomes have been regarded as important mediators in cell-to-cell communication, and their clinical utility in diagnostic applications and innovative treatment have also emerged [37, 38]. Currently, it is widely believed that exosomes show great potential in serving as biomarkers and therapeutic targets. Camussi and colleagues summarized four mechanisms of cell-to-cell communication that are mediated by exosomes [39] (Fig. 1). First, exosomes function as signaling complexes by directly stimulating target cells, which is integral, especially for the process of platelet coagulation (Fig. 1a). Moreover, neutrophils can release exosomes expressing the activated leukocyte integrin alpha M beta2 (or Mac-1), which can prompt platelet activation [39]. Second, exosomes are able to transfer receptors between cells (Fig. 1b). The receptor transfer process can occur on various cell types, such as B cells [40], platelets, endothelial cells, and tumor cells [39]. In addition, exosomes can deliver and release their protein contents within target cells (Fig. 1c). Scientists have indicated that NPC cells can release HLA class-II positive exosomes containing protein galectin 9 and/or LMP1, which has intrinsic T-cell inhibitory activity [41]. Finally, exosomes can serve to horizontally transfer genetic information mainly by relying on the transformation of miRNAs, mRNAs, or even DNAs carried by exosomes, affecting the expression in target cells (Fig. 1d). Xue et al. found a significant correlation between serum miR-93 in exosomes and clinical information, including stage and tumor size [42]. Except as a messenger in cell-to-cell communications via transferring cargo, exosomes also directly interact with extracellular matrix (ECM). The activated neutrophil-derived exosomes could bind and degrade ECM via the integrin Mac-1 and surface-bound neutrophil elastase (NE), respectively, thus causing the hallmarks of chronic obstructive pulmonary disease (COPD) and bronchopulmonary dysplasia (BPD) [43] (Fig. 1e). These above findings demonstrated remarkable versatility of exosomes in the physiological and pathological processes.

## Discovery and possible mechanism of exo-circRNAs

Based on the discovered biological characteristics of circRNAs and exosomes, increasing evidences indicates that exosomal circRNAs (exo-circRNAs) might have vital biological roles in various pathological and physiological processes. In 2015, Li et al. proved the abundance and stability of circRNAs in exosomes [7]. Besides, genome-wide analyses estimated that the abundance and circular-to-linear splicing ratio is at least 2 to 6 times higher in exosomes than in producer cells, and there are more than 1000 distinct circRNAs candidates presented in human serum exosomes [44]. In previous studies, some interesting phenomena began to attract attention. Dou et al. demonstrated that circRNAs are more abundant in exosomes than in cells and the level of circRNAs varies with different KRAS (a proto-oncogene) mutation statuses [45]. In three isogenic colon cancer cell lines, researchers discovered that circRNAs are downregulated in cell lines containing the mutant KRAS allele compared with cell lines with the wild KRAS allele. Moreover, secreted exosomes and abundant exo-circRNAs have been investigated in all these cell lines. However, the correlation of the level of circRNAs in cells and circRNAs in exosomes remains unknown, and the regulatory mechanism of exo-circRNAs still requires further study.

**Fig. 2:**
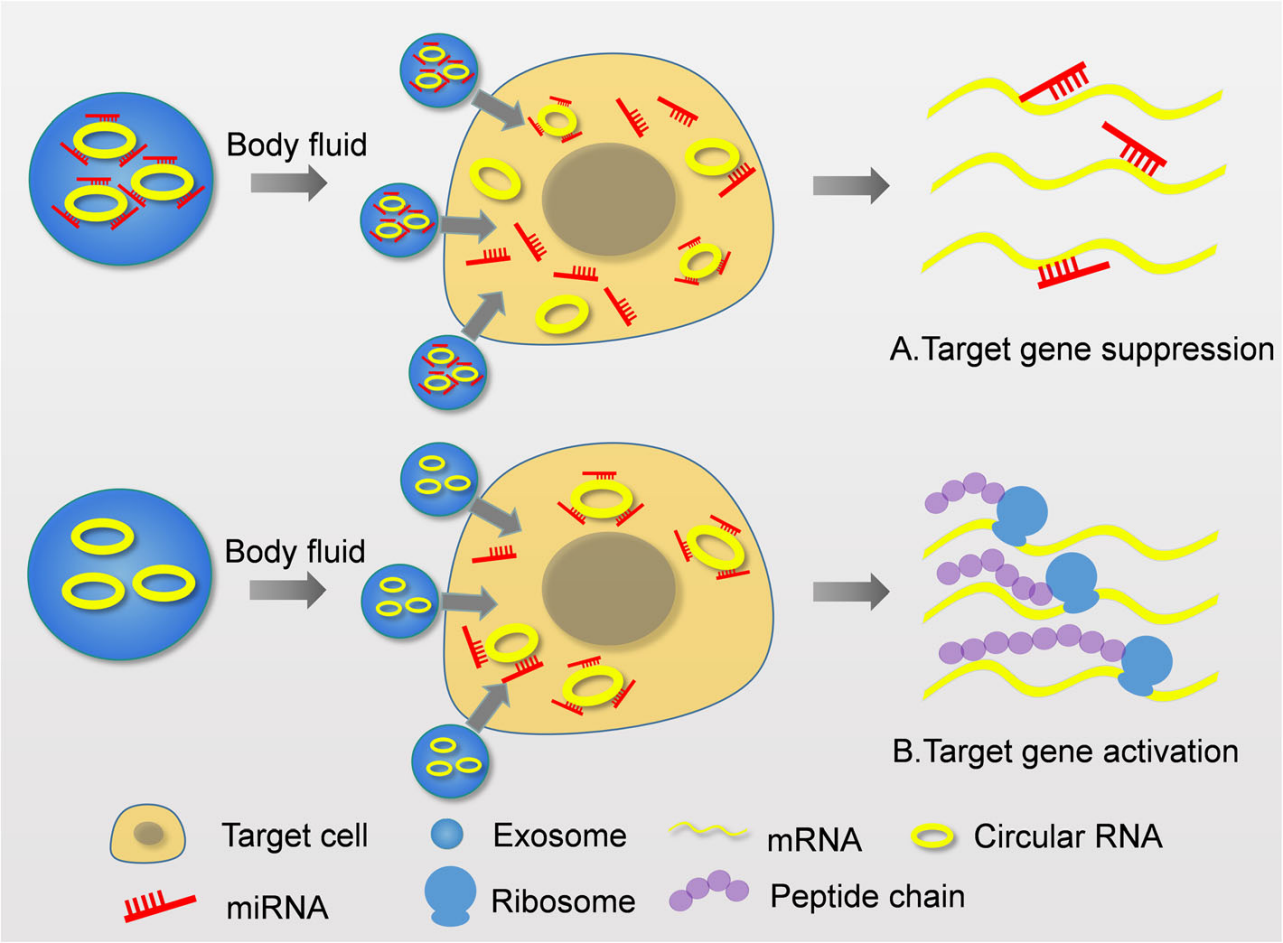
Possible mechanism of exo-circRNAs. **a** Some exo-circRNAs are bind to miRNAs in exosome. After entering target cells, miRNAs are relaesed and target genes can be silenced. **b** When exo-circRNAs are not bind to miRNAs in exosmes, they are able to sponge spcific miRNAs in target cells. As a result, target genes are acivated

To date, we suspect that miRNA sponge is still the main regulatory mechanism of exo-circRNAs (Fig. 2). The transition function of exosomes enables exo-circRNAs to regulate downstream genes with greater flexibility and universality:1) Exosomes can concentrate circRNAs which have been bound to miRNAs and then transfer them to target sites. Once circRNAs releases miRNAs, they bind to the corresponding mRNAs of target genes to silence the genes (Fig. 2a). 2) On the contrary, exosomes can carry circRNAs to the target site. Then circRNAs are desorbed and bind to miRNAs, playing the role of releasing target genes (Fig. 2b).

## Significance of exo-circRNAs in cancer

Several studies initially investigated the great potential of exosomes as biomarkers in cancer diagnosis due to the features described above. In 2008, Taylor et al. found that between the blood of patients suffering from ovarian cancer and healthy people, the expression of miRNA in exosomes differed sharply, indicating that exosomes may benefit the diagnosis of ovarian cancer [46]. Another group also claimed similar conclusions in their study on non-small-cell lung cancer (NSCLC) [47]. Except for miRNA, the expression of circRNAs in exosomes from tumors is distinct compared with that in healthy people, indicating their great clinical application value [7]. For instance, in breast cancer, the levels of carcinoembryonic antigen CEA and tumor antigen 15–3 rise continuously which are closely associated with disease stage and this is what we expect from exosomes [48]. Although so many studies have been conducted, whether exosomes are precise and useful for diagnosis is still uncertain. More clinical experiments are needed to be done.

With respect to therapy, some scientists claimed that exosomes can also be used as vaccines or delivery system. Exosomes from B lymphoma cells have been proven to be rich in HSP70 and HSP90, thus improving anti-tumor immunity [49]. Taking advantage of exosomes, scientists are able to target drugs to tumor cells. Halda and colleagues demonstrated that exosomes could increase the therapeutic index of doxorubicin (DOX). Exosomal doxorubicin (exoDOX) avoids heart toxicity by partially limiting the crossing of DOX through myocardial endothelial cells [50]. Another group showed that bovine milk can serve as a scalable source of exosomes that can act as carriers for chemotherapeutic/chemopreventive agents. Compared to free drugs in cell culture studies, drug-loaded exosomes show much higher efficacy against lung tumor xenografts in vivo [51].

So far, many studies have examined the relationship between exosomes and miRNAs, mRNAs, etc., and circRNAs as a latent found nucleic acid molecule, their role in exosomes is being explored by more and more researchers. Because they are located in the exosomes, exo-circRNAs are given the characteristic of transferable targeting ability, as well as the original biological functions of circRNAs, thus differing from the traditional endocrine circulating RNAs.

### The biological roles of exo-circRNAs in cancers

**Fig. 3:**
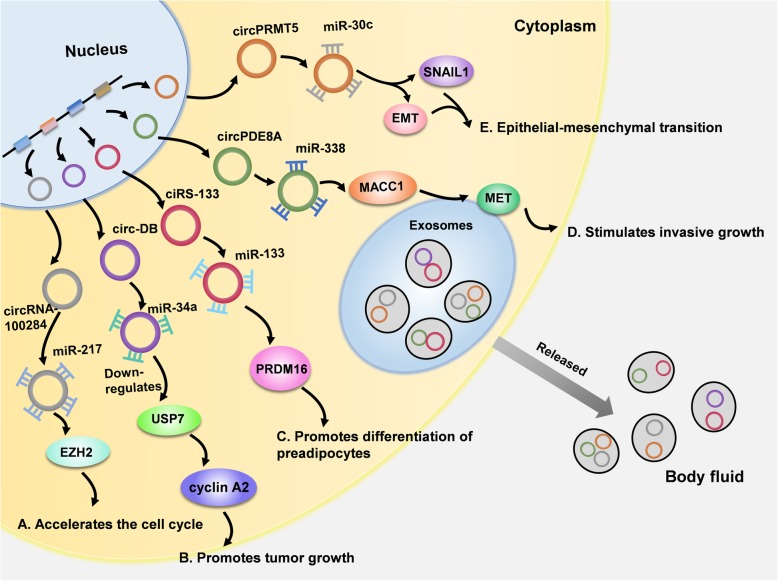
Exo-circRNAs play a crucial role via their sponging function and can be secreted into body fluid in tumors. **a** In malignant L-02 cells, circRNA-100284 can bind to miR-217, which gives rise to EZH2 and contributes to the abnormal proliferation of liver cells. EZH2 can be transported to other cells and influence their biological behavior. **b** Circ-DB from exosomes functions by downregulating expression of miR-34a and upregulating level of both USP7 and cyclin A2. Then the growth of HCC is accelerated. **c** The ciRS-133/miR-133/PRDM16 signaling pathway is important for differentiation of preadipocytes. The ciRS-133 can upregulate level of PRDM by reducing miR-133 and promote the differentiation process. **d** It has been proven that there is exosome-mediated circRNAs communication in pancreatic ductal adenocarcinoma (PDAC). Circ-PDE8A shows high expression in PDAC, and this molecule can act as a miR-338 sponge and promote the expression of the cancer-related genes MACC1 and MET. **e** The epithelial-mesenchymal transition (EMT) is a crucial process in carcinoma. Exo-circPRMT5 is reported to show a remarkable increase in serum and urine samples from patients who have urothelial carcinoma of the bladder (UCB). CircPRMT5 could promote UCB cell EMT by acting as a miR-30c sponge, and the downstream genes SNAIL1 and E-cadherin would be enhanced to promote cell invasiveness

All these above findings have aroused great attention to exo-circRNAs and may reveal their informational function and regulatory roles in pathological processes, especially for cancers. In general, there are mainly two hypotheses explaining the way exo-circRNAs function in cancer—intercellular messengers and circRNAs purgers. On the one hand, secreted exosomes containing exo-circRNAs can play a role via the special function of circRNAs (Fig. 3). As we mentioned above, circRNAs can serve as miRNA sponges, which is the most common function of circRNAs, and exo-circRNAs play an important role in this process. As circRNAs CDR1as binds to miR-7, Li and his colleague introduced miR-7 mimics into cells, which resulted in the downregulation of CDR1as in exosomes and the upregulation of CDR1as in cells [7]. This experiment verified certain conjecture—exo-circRNAs impact cell biological behavior by the level of miRNAs. Moreover, message transmission is an important process for the formation of tumors, and it is likely that exo-circRNAs serve as intercellular regulators in the process of carcinogenesis.

Arsenic is a toxic metalloid that can cause tumors in the lungs, skin and bladder under long-term exposure [52, 53]. Recently, researchers have revealed that circRNA-100284 is upregulated in malignant L-02 cells (a kind of normal human liver cell) induced by arsenite and accelerates the cell cycle and cell proliferation. Furthermore, circRNA-100284 can act as a miRNA sponge of miR-217—a tumor suppressor involved in many carcinomas, including hepatocellular cancer (HCC) [54] (Fig. 3a). Consequently, this process stimulates the downstream signal pathway and gives rise to the increase of enhancer of zeste homolog 2 (EZH2) and cyclin-D1 and lead to the abnormal proliferation of liver cells. To promote carcinogenesis, circRNA-100284 is released in exosomes from malignant transformed cells and transferred into neighboring normal cells [55]. In addition, it was observed that some exo-circRNAs derived from adipose tissues can affect the deubiquitination in HCC. Among the patients with higher body fat rate, more exo-circ-deubiquitination (circ-DB) exists. Then they proved that the circ-DB activates USP7 in HCC cells by reducing level of miR-34a. As a result, the circ-DB/miR-34a/USP7/CyclinA2 signaling pathway was found, by which the exo-circRNAs promoting cancer growth and suppressing damage to DNA [56] (Fig. 3b).

While in another gastric cancer model, one of exo-circRNAs in plasma named ciRS-133 showed close correlation with browning of white adipose tissue (WAT) and cancer-associated cachexia. After delivered to preadipocytes, ciRS-133 lower the expression of miR-133 and activates PRDM16, and the differentiation of preadipocytes into brown-like cells is accelerated (Fig. 3c). Moreover, they also founded that knockdown of ciRS-133 can prevent the tumor-implanted mice from suffering from cancer-related cachexia, indicating the importance of exo-circRNAs in the pathologic process [57].

Pancreatic ductal adenocarcinoma (PDAC) is one of the most aggressive and deadly forms of carcinomas with a low 5-year survival assembly rate of 5%, which results from a high risk of metastasis and recurrence [58–61]. Nevertheless, researchers have made a breakthrough in exosome-mediated circRNAs communication in PDAC. According to microarray analysis, circ-PDE8A is a highly expressed circRNAs in PDAC. Circ-PDE8A can bind to miR-338 and act on its target gene metastasis-associated in colon cancer-1(MACC1), which is a key regulator of MET—one of the most common oncogenes in epithelial cancers including PDAC. That is, circ-PDE8A mediates the pathological process of PDAC via the miR-338/MACC1/MET pathway [62] (Fig. 3d). In further, researchers have proven that the level of circ-PDE8A is extremely high in the serum exosomes of PDAC patients, which indicates that exo-circ-PDE8A enhances tumor invasion through exosome-mediated communication.

Pathological epithelial-mesenchymal transition (EMT) is essential in tumor development and involves the process of transforming epithelial cells into mesenchymal cells with migration ability. Once the factors of the signal process are stimulated improperly, normal cells are likely to become more drug-resistant, and angiogenesis can be activated to form a tumor microenvironment [63]. A recent study indicated that circPRMT5 was upregulated in serum and urine exosomes from urothelial carcinoma of the bladder (UCB) patients. Further investigation proved that circPRMT5 could promote UCB cell EMT by acting as a miR-30c sponge, and as a result, the expression of its target genes SNAIL1 and E-cadherin would be enhanced, which enable the cells to be more invasive [64] (Fig. 3e).

CircRNAs are widely expressed in human tissues, including blood cells [65, 66]. In a recent study, researchers extracted exosomes from platelets and found that circRNAs are selectively packaged and released into exosomes. Because platelets take part in various physiological processes, such as blood coagulation, inflammation and neoplasm metastasis, exo-circRNAs can be transported to the whole body to play a corresponding regulatory role [67].

On the other hand, we are acquainted with circRNAs biogenesis and function, although we still know little about their degradation and metabolism. Studies have shown that circRNAs show constant resistance to enzymes and have a half-life that can be longer than 48 h [5, 11, 22]. Given the richness and stability of circRNAs, researchers assume that the build-up of circRNAs can be toxic and exosomes transfer circRNAs from cells to enforce circRNAs clearance by means of exocytosis. Intriguingly, the expression levels of circRNAs are obviously higher than their corresponding mRNA levels in exosomes, while the expression levels of mRNAs in exosomes are lower than those in cells, which indicates that circRNAs enter exosomes for their clearance [68]. In conclusion, the research of exo-circRNAs has been conducted for several years, unveiling the mystery of exo-circRNAs and leaving many questions to be solved in further studies.

### The potential application of exo-circRNAs in anticancer therapy

**Fig. 4:**
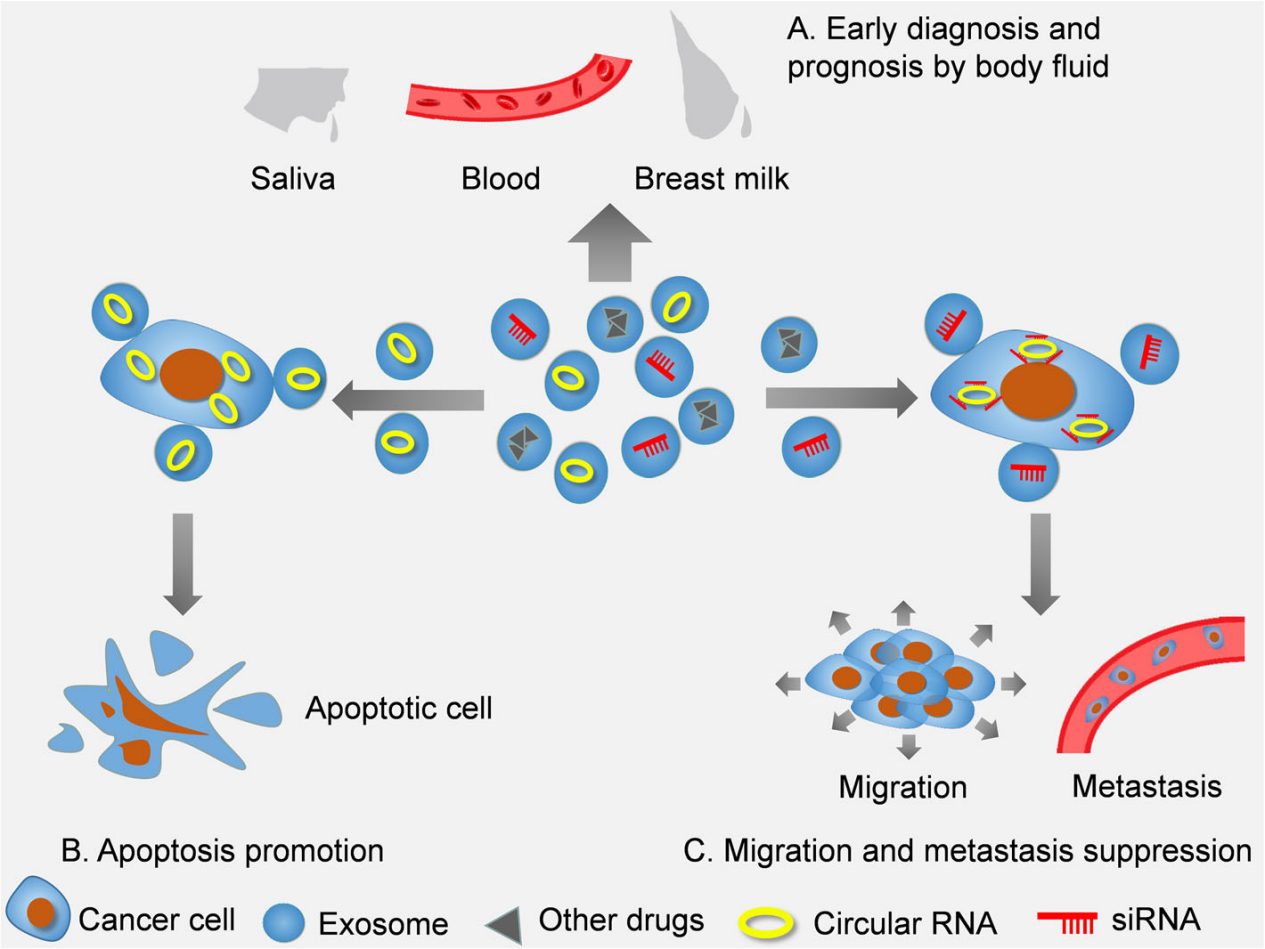
Possible application of exo-circRNAs in anticancer. **a** Through the examination of exo-circRNAs in body fluids, such as saliva, blood, and breast milk, we are likely to help identify and predict the outcome of cancer. **b** An increased number of circRNAs can lead to a higher apoptosis rate. **c** Specific drugs and siRNAs that inhibit the functions of some circRNAs tend to suppress the migration and metastasis of cancer

Currently, clinical progress has been made in the early diagnosis, surgical methods, radiotherapy and chemotherapy of kinds of cancers. However, the early symptoms of some tumors are not typical, and the final diagnosis often requires a biopsy, which can be painful and complicated for the patients. There is still a lack of rapid, accurate and noninvasive early diagnostic biomarkers in clinical uses. Besides, regular follow-up is necessary for cancer patients who suffer from operation or chemoradiotherapy, and the examination of tumor markers is indispensable—similar to the function of the index AFP (alpha fetoprotein) in liver cancer. However, there are still many carcinomas lacking an authoritative index, and many researchers are expecting to find some in circRNAs. Thus far, some studies have indicated that microRNAs and long noncoding RNAs can be used as biomarkers in tumors [69, 70]. However, the characteristics of circRNAs make these molecules a better choice to mark diseases because of their closed loop structure and insensitivity to RNase. Compared with the 48 h half-life of most circRNAs, the average half-life of microRNAs is usually less than 10 h [71]. Consequently, the potential for circRNAs to be putative biomarkers in clinically relevant samples is being explored widely. It is now clear that exosomes can be perfect carriers for circRNAs, which are abundant, conserved and stable. Tumor-generated exo-circRNAs can be secreted into blood, saliva, urine, cerebrospinal fluid, milk and many other body fluids to have an impact on diagnosis, tumor cell apoptosis and suppression of metastasis etc. [72] (Fig. 4). For instance, we have illustrated that exo-circ-PDE8A plays a significant role in the malignancy of pancreatic cancer. Researchers have discovered that the expression level of circ-PDE8A in the blood was much higher in the pancreatic cancer model than in the NC group. Then, they extracted blood exosomes from PDAC patients, and further analysis showed that the high expression of exo-circPDE8A was closely linked with duodenal invasion, vascular invasion and TNM stage [62]. Furthermore, survival analysis also proved that the high expression of exo-circPDE8A was a risk factor and that people with lower expression of exo-circPDE8A enjoy a longer expectancy. In this case, we can use exo-circPDE8A in both early diagnosis and prognosis to determine whether the patient is continuously being invaded by cancer (Fig. 4a).

From the first indentification of the abundance of circRNAs in exosomes in 2015, researchers found that circRNAs transferred by exosomes from producer cells may play a part in the biological activity of the recipient cells [7]. After being relocated to recipient cells, the circRNAs named CDR1as suppresses growth by inhibiting miR-7. This report provides a novel direction for the treatment of diseases, especially cancers. Numerous investigations have reported various signaling pathways involving circRNAs in cancer cells. Exosomes, regarded as vehicles of circRNAs, have the potential to upregulate the content of circRNAs in cells, which is likely to suppress malignant behavior in cancer. In a study of Liu and colleagues, a synthetic circular RNA named scRNA21 that was designed to function as a sponge of miR-21, was successfully formed in vitro [73]. After the transfection of scRNA21 into three kinds of gastric carcinoma (GC) cells, it was found that scRNA21 induced increased apoptosis compared to the control group in all three GC cell lines (Fig. 4b).

In addition, as exosomes have various cargoes, there is a possibility of taking advantage of exosomes to deliver therapeutic drugs to cancer cells. Considering that many circRNAs were confirmed to promote the progression of cancer, exosomes carrying drugs, such as specially designed small interfering RNAs (siRNAs) that target specific circRNAs, can help lower the expression of negative circRNAs in cancer cells. As a result, these molecules can indirectly inhibit the harm induced by circRNAs through sponging miRNAs and advancing the expression of antioncogenes. For example, once the circRNA-ACAP2 and circCCDC66 were inhibited by transfecting siRNAs in colon cancer cells, the decreased cell proliferation, migration and invasion rate could be observed compared with that of the control and NC groups [5, 74] (Fig. 4c). In addition, similar studies can be found in osteosarcoma [75–77], gastric cancer [78], pancreatic cancer [79], cervical cancer [80–82], oral cancer [83], gallbladder cancer [84], and breast cancer [85].

## Perspectives

When first discovered by scientists, circRNAs was initially regarded as an error of transcription. The biological functions of circRNAs have been proven in the last 5 years, among which the sponging of miRNA has attracted the attention of most researchers, and numerous pathways have been identified. As a result, these molecules may be a potential target of therapy. In addition, due to the closed loop structure, circRNAs are insensitive to exonucleases, are more stable in tissues and plasma and are confirmed to be expressed differently in neoplasm tissues and normal adjacent tissues. These findings, in turn, show the possibility of biomarkers in early diagnosis and prognosis in diseases, especially cancer.

Exosomes serve as vehicles carrying proteins, miRNAs, mRNAs, DNA, and circRNAs. These molecules play vital roles in cell-to-cell communication and are also recognized as possible biomarkers for their detective features. The exo-circRNAs are circRNAs delivered by exosomes and can be found in various kinds of body fluids. Many studies have already highlighted the possible application in diagnosis as well as novel therapy. Despite the promising prospects, many difficulties must be overcome. Although more articles have emerged recently, further studies are lacking compared to studies on mRNAs and miRNAs, which means before application to clinics, we should have a more accurate understanding of these molecules. In our view, the exo-circRNAs would be one of the most popular issues in the future, and there would be enough theoretical investigations supporting its clinical application.

## Abbreviations

AFP: Alpha fetoprotein
BPD: Bronchopulmonary dysplasia
ceRNAs: Competitive endogenous RNAs
circ-DB: Exo-circ-deubiquitination
circRNAs: Circular RNAs
ciRNAs: Intronic circRNAs
COPD: Chronic obstructive pulmonary disease
DOX: Doxorubicin
ecircRNAs: Exonic circRNAs
ECM: Extracellular matrix
EIciRNAs: Exonic-intronic circRNAs
EMT: Epithelial-mesenchymal transition
ESCRT: Endosomal Sorting Complex Required for Transport.
EVs: Extracellular vehicles
exo-circRNAs: Exosomal circRNAs
exoDOX: Exosomal doxorubicin
EZH2: Enhancer of zeste homolog 2
GC: Gastric carcinoma
HCC: Hepatocellular cancer
ILVs: Intraluminal vesicles
MACC1: Metastasis-associated in colon cancer-1
MREs: miRNA response elements
MVBs: Multivesicular bodies
NE: Neutrophil elastase
NSCLC: Non-small-cell lung cancer
PDAC: Pancreatic ductal adenocarcinoma
RNA-Seq: RNA sequencing
siRNAs: Small interfering RNAs
SRY: Sex-determining region Y
UCB: Urothelial carcinoma of the bladder
Vps 4: Vacuolar protein sorting 4
WAT: White adipose tissue
